# Nattokinase attenuates bisphenol A or gamma irradiation-mediated hepatic and neural toxicity by activation of Nrf2 and suppression of inflammatory mediators in rats

**DOI:** 10.1007/s11356-022-21126-9

**Published:** 2022-06-01

**Authors:** Mustafa M. M. Elbakry, Somaya Z. Mansour, Hamed Helal, Esraa S. A. Ahmed

**Affiliations:** 1 Biochemistry Department, Faculty of Science, Ain Shams University, Cairo, Egypt; 2 Radiation Biology Research, National Center for Radiation Research and Technology, Egyptian Atomic Energy Authority, Nasr City, Cairo, 11787 Egypt; 3 Zoology Department, Faculty of Science, Al-Azhar University, Cairo, Egypt

**Keywords:** Nattokinase, Bisphenol A, Gamma irradiation, NF-κB/Nrf2/HO-1, Oxidative stress, Inflammatory response

## Abstract

Nattokinase (NK), a protease enzyme produced by *Bacillus subtilis*, has various biological effects such as lipid-lowering activity, antihypertensive, antiplatelet/anticoagulant, and neuroprotective effects. Exposure to environmental toxicants such as bisphenol A (BPA) or γ-radiation (IR) causes multi-organ toxicity through several mechanisms such as impairment of oxidative status, signaling pathways, and hepatic and neuronal functions as well as disruption of the inflammatory responses. Therefore, this study is designed to evaluate the ameliorative effect of NK against BPA- or IR-induced liver and brain damage in rats. Serum ammonia level and liver function tests were measured in addition to brain oxidative stress markers, amyloid-beta, tau protein, and neuroinflammatory mediators. Moreover, relative quantification of brain nuclear factor-erythroid 2-related factor-2 (Nrf2)/heme oxygenase-1 (HO-1) genes, as well as apoptotic markers in brain tissue, was carried out in addition to histopathological examination. The results showed that NK improved liver functions, impaired oxidative status, the cholinergic deficits, and minified the misfolded proteins aggregates. Furthermore, NK alleviated the neuroinflammation via modulating NF-κB/Nrf2/HO-1 pathway and glial cell activation in addition to their antiapoptotic effect. Collectively, the current results revealed the protective effect of NK against hepatic and neurotoxicity derived from BPA or IR.

## Introduction

The proven efficacy of medicinal plants in treating various diseases and their safe impact on human health elevates their consumption and use in the manufacture of drugs globally (Michel et al. 2020; Ullah et al. 2020). Various medicinal plants such as *Phoenix dactylifera* have anti-inflammatory, anti-hepatotoxicity, and anti-neurotoxicity (El-far et al. 2019). Others have anti-oxidative and anti-apoptotic effects against toxicity such as thymoquinone (Hassan et al. 2019). Nattokinase (NK) is an alkaline protease enzyme produced by *Bacillus subtilis* during the fermentation of typical Japanese food natto. NK has been used in the prevention of cardiovascular diseases due to its potent fibrinolytic and antithrombotic effects (Huang et al. 2021). Notably, NK possesses a potent lipid-lowering activity, antihypertensive, and neuroprotective effects and is considered a valuable natural product with various pharmaceutical, health, and medical applications (Pham et al. 2020; Zhang et al. 2020). Furthermore, previous studies showed that NK offers multiple health benefits such as the treatment of hemorrhoids, diabetes, muscle spasms, chronic inflammation, cardiovascular diseases, Alzheimer’s disease, stroke, etc. (Nguyen and Nguyen 2020; Takagaki et al. 2020).

Excessive exposure to high environmental temperatures (Abdelnour et al. 2019) and a wide range of environmental pollutants such as toxic chemicals, UV radiation, heavy metal ions, and others elevates the harmful consequences on human health (Alkhalf and Khalifa 2018) via activation of the inflammatory signaling in tissues. Radiation exposure either through natural, radiotherapy, medical, or accidental exposure is associated with various complications like immunosuppression, endocrine dysfunction, and organ injury (Hanedan Uslu et al. 2019). A cascade of radiation-induced tissue injury in living organisms initiates after exposure to relatively high-dose radiation which may cause the death of the organism within a short period due to acute effects (Chiba et al. 2002). The early response of the immune system to ionizing radiation is via inflammatory response and secretion of many inflammatory mediators (Najafi et al. 2018). Ionizing radiation triggers a state of oxidative stress and production of reactive oxygen species (ROS) through radiolysis of water molecules, the main component of tissues and consequently impairs various biochemical processes and molecular signaling pathways (Wei et al. 2019; Tiziana Cervelli et al. 2021).

Considering the wide application of bisphenol A (BPA) in the manufacture of many consumable foods and beverage containers, toys, and medical equipment, it is considered a prevalent environmental and industrial toxin (An et al. 2021). The prolonged exposure and excessive use of BPA in daily life through environmental pollution, ingestion, inhalation, or dermal contact boost detrimental impacts on human health (Thoene et al. 2017). Previous studies revealed that even low doses of BPA induce mutagenicity, hepatotoxicity, neurotoxicity, and immunotoxicity by inducing oxidative stress (Basit et al. 2020; Eweda et al. 2020).

Moreover, there is a relationship between the functional status of the liver and brain known as hepatic encephalopathy (HE) resulting from insufficiency of the liver to remove toxins from the blood leading to brain dysfunction. It is manifested via a wide spectrum of neurologic abnormalities ranging from increased circulating neurotoxin levels (such as ammonia), impaired neurotransmission, changes in brain energy metabolism, and systemic inflammatory response (Jiménez-Torres et al. 2021). Collectively, this study was designed to evaluate the possible ameliorative effect of nattokinase on hepatic and neurotoxicity as well as the histopathological changes induced in rats either by BPA or γ-irradiation.

## Materials and methods

### Chemicals

Bisphenol A (BPA) was purchased from Merck KGaA, Darmstadt, Germany, CAS Number: 80–05-7, Cat. No.: 803546. Nattokinase (NK) was purchased from Doctor’s Best (USA), Item Code: 286,737, UPC: 75,395,000,253.

### Ethics statement

This experiment was carried out according to the Guide for the Care and Use of Laboratory Animals published by the National Institutes of Health (No. 85:23, 1996) and in compliance with the principles and guidelines of the animal care committee of the National Center for Radiation Research & Technology (NCRRT), Cairo, Egypt. Every attempt was made to keep animal suffering to a minimum.

### Animals

Forty-eight adult female Swiss albino rats (120–150 g) were obtained from the animal farm of the Egyptian Holding Company for Biological Products and Vaccines (VACSERA), Cairo, Egypt. The rats were housed in a conditioned atmosphere (20–22 °C) in suitable cages and maintained on tap water and a standard diet (Clarke et al. 1977).

### Radiation facility

Rats were exposed to whole-body gamma-radiation (IR) at The National Center for Radiation Research and Technology, Atomic Energy Authority, Cairo, Egypt, using the cesium-137 biological irradiator source (gamma-cell-40), Atomic Energy of Canada Limited (Chalk River, ON, Canada).

### Experimental design

Rats were randomly divided into 6 equal groups as follows:

- Control group: Healthy control animals (vehicle group): Rats were given orally corn oil by gavage.
- NK group: Rats were orally gavaged with NK at a dose of 720 FU/kg BW/day for 5 weeks according to Fadl et al. (2013).
- BPA group: Rats were injected intraperitoneally with BPA at a dose of 100 mg/kg BW/day which was dissolved in corn oil for 5 weeks according to Bilgi et al. (2019).
- BPA + NK group: Rats were injected with BPA as in group 3 and treated orally gavaged with NK as in group 2.
- IR group: Rats were exposed to whole-body IR at a dose of 3 Gy/week for 4 weeks according to Trivedi et al. (2012).
- IR + NK group: Rats were exposed to whole-body IR as in group 5 and were treated with NK as in group 2.

### Collection of samples

After the last dose of NK, rats fasted overnight before scarification under light ether anesthesia. Blood was drawn from the vena cava and the sera were separated by centrifugation for 10 min at 3000 × g for biochemical parameter estimation. Following blood sample collection, the liver and the brain were excised, cleaned, and washed with saline (0.9% sodium chloride). Some parts of the brain were stored at − 80 °C for biochemical parameter assessment, while the other parts of tissues (brain and liver) were rinsed in 10% neutralized formalin for histopathological examination.

### Biochemical estimations

Using Thermo Spectronic Helios Delta Visible spectrophotometer, the levels of ammonia and total protein were determined according to the method described by Konitzer and Voigt (1963) and Gornall et al. (1949), respectively. Also, liver alanine transaminase, aspartate transaminase, and alkaline phosphatase activities were measured using commercial kits from Bio-diagnostic, Egypt, according to the previously described methods (Reitman and Frankel 1957; Moss 1982). The oxidative stress markers in the brain tissues malondialdehyde (MDA) and glutathione (GSH) levels were estimated according to previous studies of Ohkawa et al. (1979) and Beutler et al. (1963), respectively.

### Estimation of brain inflammatory markers

The levels of interleukin-6 (IL-6) were measured in brain tissue using an enzyme-linked immunosorbent assay (ELISA) kit obtained from Thermo Fisher Scientific Inc. (USA), catalog no. ERA32RB. Also, interleukin-10 (IL-10) levels were measured in brain tissue using an ELISA kit purchased from Abcam (UK), catalog no. ab100765. TECAN (Magellan™) was used as a plate reader and data analysis software for ELISA.

### Estimation of neurotoxicity markers: amyloid β, tau proteins, and acetylcholine

Amyloid β-protein (Aβ) level was determined by using an ELISA kit purchased from MyBiosource, Inc. (USA), catalog no. MBS726579; phosphorylated tau protein (p-tau) level was measured using an ELISA kit purchased from MyBiosource, Inc. (USA), catalog no. MBS725098. Moreover, acetylcholine level was measured using an ELISA kit purchased from MyBiosource, Inc. (USA), catalog no. MBS728879, according to the manufacturer’s instruction. Readings and analysis of ELISA plate results were carried out using TECAN (Magellan™).

### Western blotting

Brain tissue homogenate was prepared, and western blot analysis was carried out as previously stated by Chen et al. (2005) and Omar et al. (2009) using anti-beta actin antibody (ab8227), anti-GFAP antibody (ab7260), and anti-NF-kB p65 (ab76302) obtained from Abcam (UK). Immunoblot visualization was done using the Amersham detection kit, according to the manufacturer’s protocols, followed by exposure to X-ray film. Auto-radiograms were scanned for quantification of protein levels using a scanning laser densitometer (Biomed Instrument Inc., USA) using Image J analysis of the autoradiograms. Calculation of results was performed following normalization by housekeeping protein (β-actin).

### Quantitative real-time polymerase chain reaction

Total RNA was isolated from the brain tissue homogenates according to instructions of the manufacturer using RNeasy Micro Kit purchased from QIAGEN (Germany), catalog no. 74004. The extracted RNA was reversely transcribed to complementary DNA using an RT-PCR kit (Applied Biosystem, Waltham, MA, USA). Quantitative RT-PCR of Nrf2, HO-1, Bcl-2, and caspase-3 was performed using SYBR Green PCR Master Mix (Qiagen, Germany) as described by the manufacturer and glyceraldehyde 3-phosphate dehydrogenase (GAPDH) was used as a housekeeping gene. The primer sequences are described in Table 1. The amplification and analysis were performed using an Applied Biosystems with software version 3.1 (StepOne™, USA). The relative expression of target genes was obtained using the ΔΔCt method (Livak and Schmittgen 2001).

**Table 1:** Primers sequence

Target gene	Primer sequence
Nrf2	F: 5ʹ-CACTCTGTGGAGTCTTCCATTT-3ʹ R: 5ʹ-GAATGTGTTGGCTGTGCTTTAG-3ʹ
HO-1	F: 5ʹ-GATGGCCTCCTTGTACCATATC-3ʹ R: 5ʹ-AGCTCCTCAGGGAAGTAGAG-3ʹ
Bcl-2	F: 5ʹ-GGAGGATTGTGGCCTTCTTT-3ʹ R: 5ʹ-GTCATCCACAGAGCGATGTT-3ʹ
Caspase-3	F: 5ʹ-CTGACTGGAAAGCCGAAACT-3ʹ R: 5ʹ-GTTCCACTGTCTGTCTCAATACC-3ʹ
GAPDH	F: 5ʹ-ACTCCCATTCTTCCACCTTTG-3ʹ R: 5ʹ-CCCTGTTGCTGTAGCCATATT-3ʹ

### Detection of apoptosis by annexin V staining

For the quantitative evaluation of apoptosis, the annexin V-fluorescein isothiocyanate (FITC) and PI dual staining techniques were employed. Briefly, the peripheral blood mononuclear cells (PBMC) were isolated from the peripheral blood using the method of Böyum (1968). Cells were collected and the suspension was made in the binding buffer (Becton Dickinson). Subsequently, the cells (approximately 4 × 10^5^ cells) were stained using a BD Pharmingen FITC Annexin V apoptosis detection kit (BD Bioscience) for 15 min in the dark according to the manufacturer’s instructions. After that, the samples were analyzed within 1 h using a BD Accuri Tm C6 Plus flow cytometer, and the degree of apoptosis was quantified as a percentage of the annexin V-positive and PI-negative (annexin V + /PI −) cells.

### Histopathological assessment

Liver and brain tissue specimens were fixed in 10% formalin saline, then trimmed off, washed, and dehydrated in ascending grades of alcohol. The dehydrated specimens were then cleared in xylene, embedded in paraffin blocks, and sectioned at 4–6 µm thick. The obtained tissue sections were deparaffinized using xylol and stained using hematoxylin and eosin (H&E) for histopathological examination using the light microscope according to Downie (1990). The frequency and severity of liver lesions were assessed semi-quantitatively as previously stated by Plaa et al. (2014) and Khafaga et al. (2019) in Table 2A. The severity of cerebral histopathological changes in terms of nuclear pyknosis and degeneration in neurons, focal gliosis, and encephalomalacia was scored from 0 to 3 as reported by Abdel-latif et al. (2018) and Khafaga et al. (2021) in Table 2B.

**Table 2:** (A) Grading system for liver lesions and (B) score system for cerebral histopathological changes

(A)		(B)	
Grade	Liver lesions	Score	Cerebral histopathological changes
None (0)	No apparent injury	0 ( −)	Normal
Mild (1)	Swelling of hepatocytes (0–25%)	1 ( +)	Mild damage
Moderate (2)	Ballooning of hepatocytes (25–50%)	2 (+ +)	Moderate damage
Severe (3)	Lipid droplets in hepatocytes (50–75%)	3 (+ + +)	Severe damage
Severe (4)	Necrosis of hepatocytes (75–100%)		

### Statistical analysis

Using one-way analysis of variance (ANOVA) followed by a post hoc test (least significant difference (LSD)), the obtained data were expressed as the mean ± SD. The level of significance between the mean values was set at *p* ≤ 0.05. Statistical Package for Social Science (SPSS) version 20 for Windows (SPSS® Chicago, IL, USA) software was used to analyze the data.

## Results

### Effect of nattokinase on ammonia level

As shown in Table 3, the intoxication with BPA and exposure to γ-IR resulted in a marked increase in the level of ammonia compared to the control group, while the oral administration of NK showed an opposite effect through a notable reduction of ammonia level.

**Table 3:** Effect of NK on ammonia level, total protein, and liver function tests in the different groups

Group parameters	Control	NK	BPA	BPA + NK	IR	IR + NK
Ammonia (μg/dL)	79.4 ± 2.2^†‡^	87.1 ± 1.6^⁎†‡^	189.2 ± 3.1^⁎‡^	103.7 ± 1.4^⁎†‡^	114.9 ± 1.7^⁎†^	94.1 ± 1.09^⁎†‡^
Total protein (g/dL)	10.4 ± 0.7^†‡^	10.3 ± 0.67^†‡^	6.35 ± 0.12^⁎‡^	9.24 ± 0.24^⁎†‡^	5.5 ± 0.21^⁎†^	8.7 ± 0.2^⁎†‡^
ALT (U/L)	13.3 ± 1.2^†‡^	14.17 ± 1.17^†‡^	71.83 ± 5^⁎‡^	34 ± 1.4^⁎†‡^	65.2 ± 5.23^⁎†^	36.8 ± 1.47^⁎†‡^
AST (U/L)	18.8 ± 1.5^†‡^	22 ± 1.41^†‡^	62.5 ± 6.72^⁎^	30.3 ± 5.4^⁎†‡^	64.17 ± 1.47^⁎^	35.8 ± 1.9^⁎†‡^
ALP (U/L)	115.8 ± 3.1^†‡^	119.4 ± 3.96^†‡^	297.5 ± 13.8^⁎‡^	195.2 ± 2.7^⁎†‡^	235.3 ± 7.54^⁎†^	156.1 ± 9.24^⁎†‡^

Values were expressed as means ± SD (*n* = 6). ^⁎^, ^†^, and.^‡^ denote significant change at *p* ≤ 0.05 versus control, BPA, and IR groups, respectively

### Effect of nattokinase on parameters of liver

The results of the present study showed that the intoxication with BPA and exposure to γ-IR induced extraordinarily liver damage which was evident through impaired liver functions accompanied by extensive leakage of the hepatic enzymes (ALT, AST, and ALP) to the bloodstream, and this was confirmed with the histopathological examination of liver tissue. Meanwhile, treatment with NK markedly ameliorates the increment in these serum liver function parameters as illustrated in Table 3.

Additionally, a notable decline in the total protein levels was observed in BPA-intoxicated rats and gamma-irradiated rats. On the other hand, oral supplementation with NK significantly increased total protein levels (Table 3).

### *Effect of NK treatment on oxidative stress biomarkers in *brain tissue

**Fig. 1:**
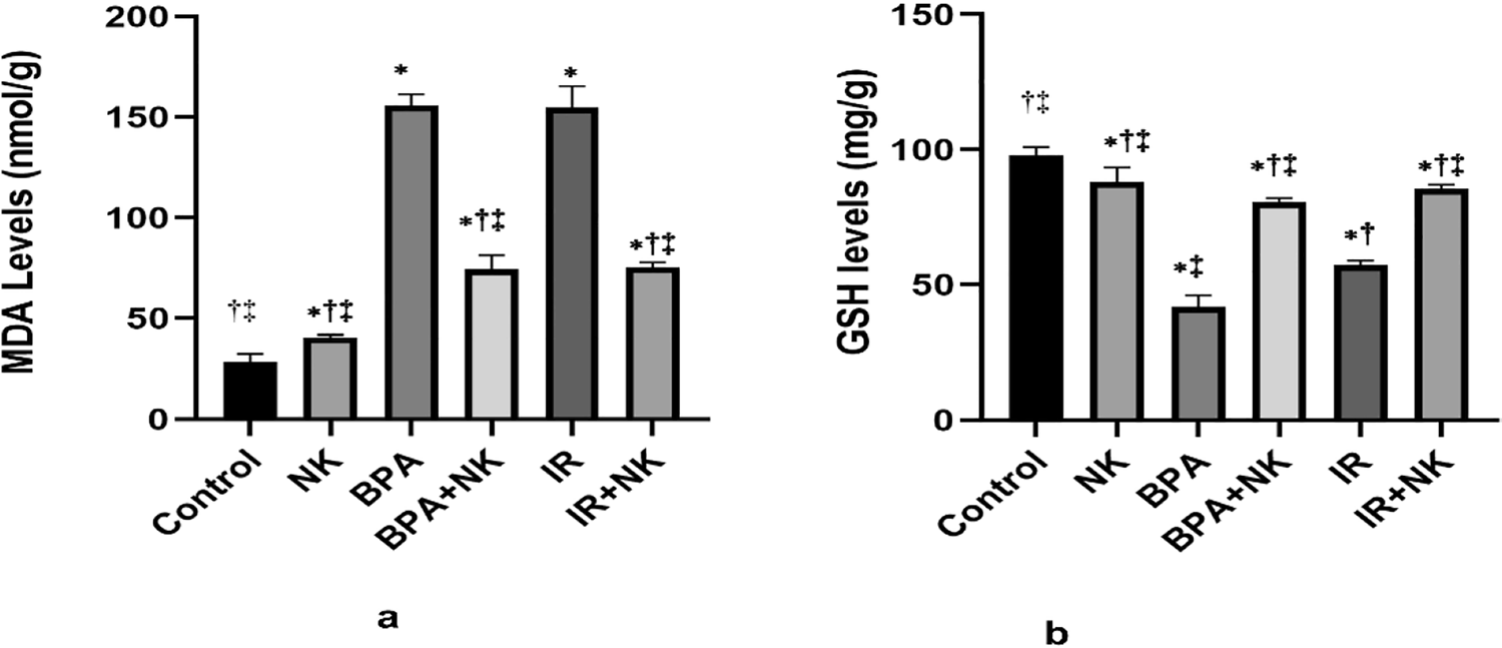
Effects of NK on oxidative stress parameters, MDA (**a**) and GSH (**b**) in the brain tissues. Data are presented as the means ± SD. ⁎, †, and ‡ denote significant change at *p* ≤ 0.05 versus control, BPA, and IR groups, respectively

The data illustrated in Fig. 1 shows a significant increase in the levels of lipid peroxidation (MDA) together with a marked decline in the levels of GSH in the brain tissues of rats injected with BPA or exposed to γ-irradiation in comparison to control rats. However, upon treatment with NK, the GSH levels were ameliorated along with a notable reduction of lipid peroxidation.

### Effect of NK on the amyloid-β, tau protein, and acetylcholine

**Fig. 2:**
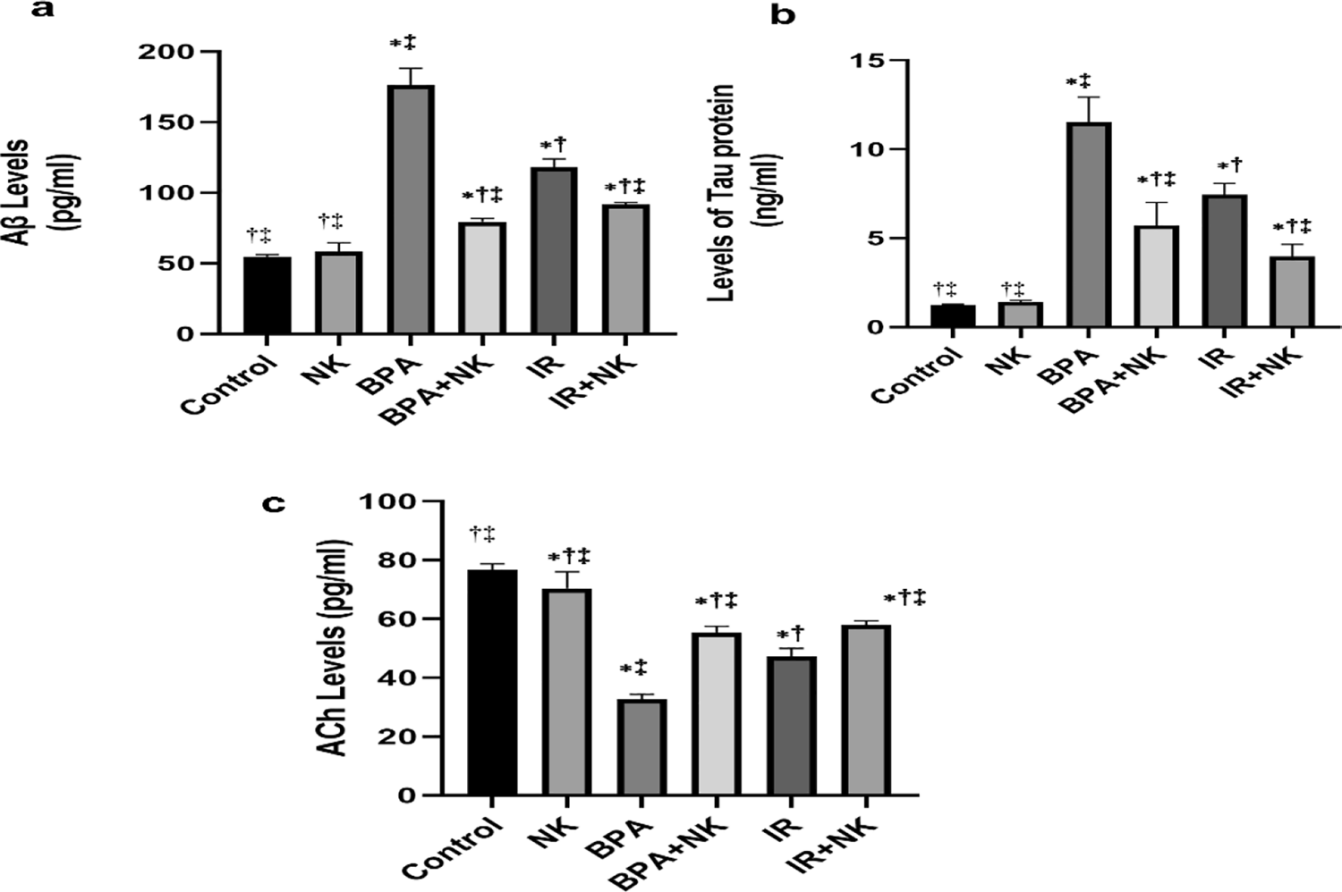
Effects of NK on Aβ (**a**), tau protein (**b**), and ACh (**c**) in the brain tissues. Data are presented as the means ± SD. ⁎, †, and ‡ denote significant change at *p* ≤ 0.05 versus control, BPA, and IR groups, respectively

Alteration in the levels of the amyloid-β (Aβ) and p-tau in the brain tissues is illustrated in Fig. 2, which indicates that both BPA intoxication and exposure to IR significantly augmented the levels of both Aβ and p-tau in the brain tissues as compared to the control group. Alternatively, supplementation with NK markedly declined the Aβ and p-tau protein aggregation in the brain tissues of rats. Besides, the current results reported a significant reduction in the levels of acetylcholine (ACh) in the brain tissues of the BPA or IR groups compared to the control. Conversely, treatment with NK significantly improved the brain’s ACh levels (Fig. 2).

### Influence of NK on inflammatory markers in the brain tissue of rats

**Fig. 3:**
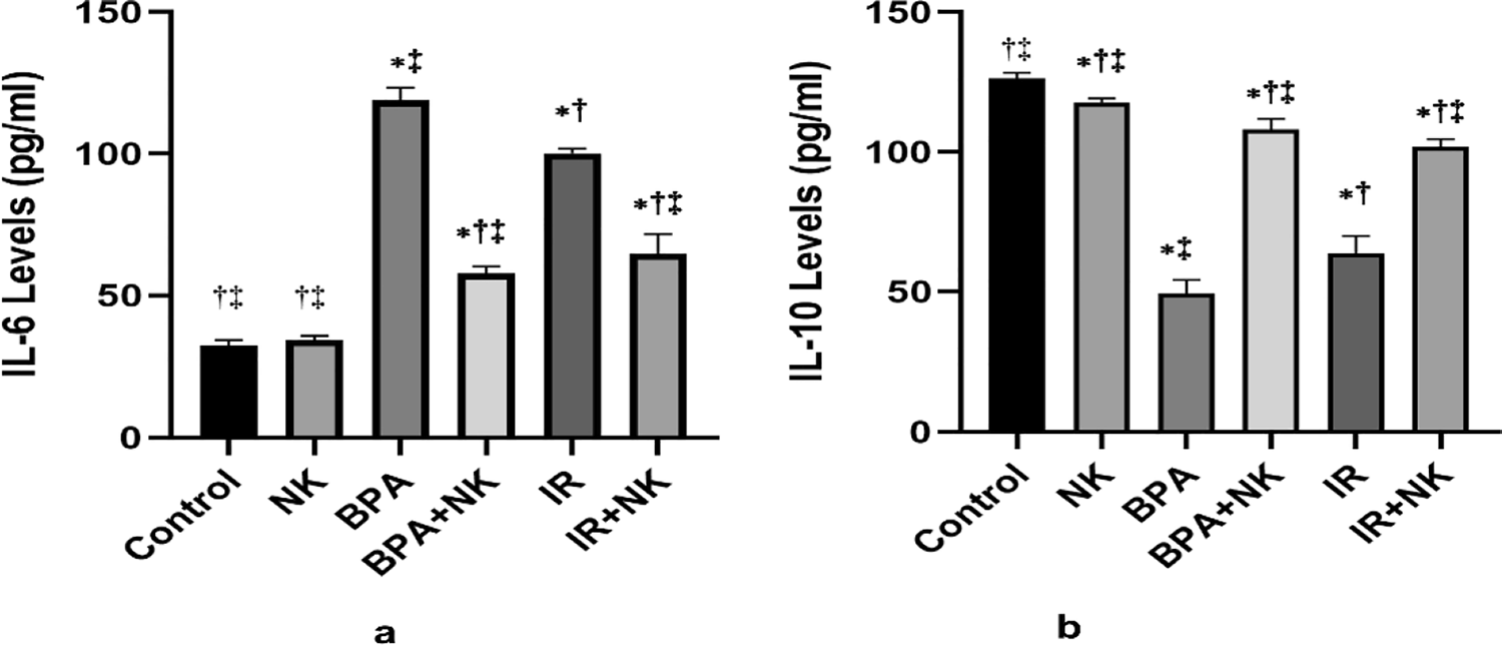
Effect of NK on neuroinflammatory cytokines, **a** IL-6 and **b** IL-10 in the brain tissues. Data are presented as the means ± SD. ⁎, †, and ‡ denote significant change at *p* ≤ 0.05 versus control, BPA, and IR groups, respectively

Inflammation is the response of tissues when stimulated or exposed to a harmful hazard such as toxins, chemicals, radiation or injured by pathogens. Both pro- and anti-inflammatory signaling pathways are involved. Furthermore, there was a significant increase in the levels of pro-inflammatory IL-6 with a concomitant decrease in the levels of the anti-inflammatory IL-10 in the brain tissues of rats in the BPA group or IR group compared to the control. Meanwhile, NK alleviated the inflammatory response via increasing the levels of IL-10 and suppressing the IL-6 (Fig. 3).

### Effect of nattokinase on NF-κB and GFAP protein expression

**Fig. 4:**
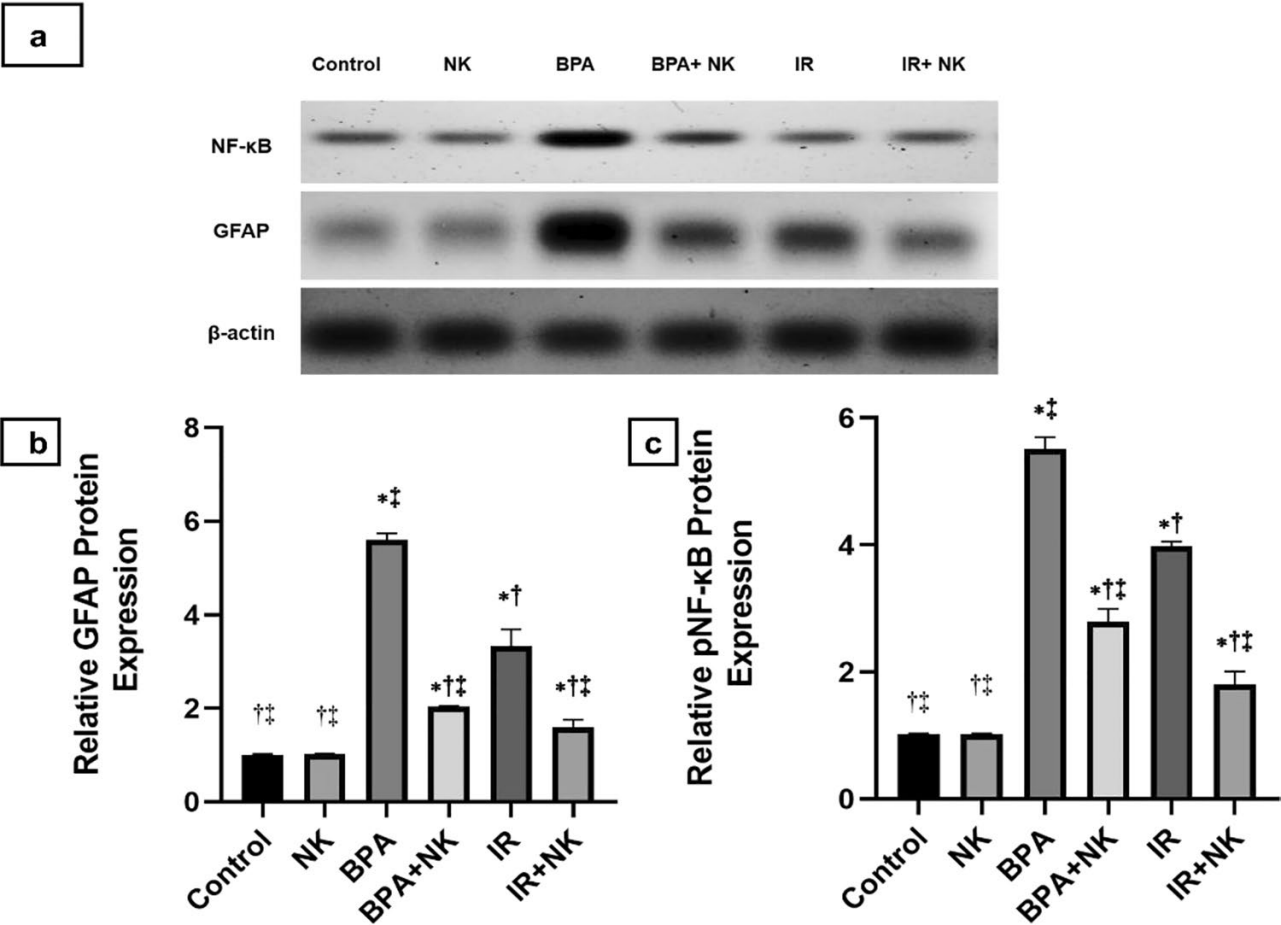
**a** Western blotting of GFAP, p-NF-κB, and β-actin. Quantitative western blotting analysis of GFAP (**b**) and p-NF-κB (**c**). Data are presented as the means ± SD. ⁎, †, and ‡ denote significant change at *p* ≤ 0.05 versus control, BPA, and IR groups, respectively

Western blot analysis was used to detect the protein levels of phosphorylated nuclear factor kappa B (p-NF-κB) and glial fibrillary acidic protein (GFAP) in the brain tissues of rats. As illustrated in Fig. 4, BPA and IR resulted in marked upregulation (*p* ≤ 0.001) of the p-NF-κB and GFAP levels as compared to the control group. Conversely, administration of NK to BPA or IR rats effectively reverted the results and caused a remarkable downregulation (*p* ≤ 0.001) in the expression levels of p-NF-κB and GFAP proteins.

### Effect of nattokinase on Nrf2 and HO-1 gene expression

**Fig. 5:**
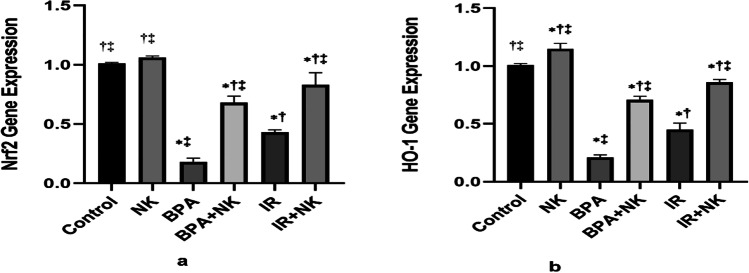
Relative quantification of Nrf2 (**a**) and HO-1 (**b**) genes in brain tissues. Data are presented as the means ± SD. ⁎, †, and ‡ denote significant change at *p* ≤ 0.05 versus control, BPA, and IR groups, respectively

Nrf2-related/heme oxygenase 1 (Nrf2/HO-1) is an important signaling pathway that plays an important role in inflammatory-derived diseases such as liver damage and neurodegenerative disease. Quantitative RT-PCR was used to measure the effect of NK on the gene expression of Nrf2 and HO-1 genes. The obtained results showed that the gene expression of Nrf2 and HO-1 in the brain tissues was downregulated upon intoxication with BPA or exposure to gamma radiation compared to the control. However, NK treatment caused a remarkable upregulation in the expression of both genes Nrf2 and HO-1 (Fig. 5).

### Influence of NK on the brain apoptosis induced by BPA or IR

**Fig. 6:**
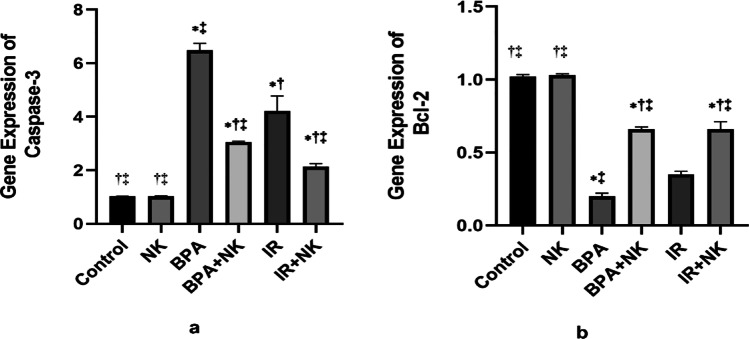
Quantitative RT-PCR analysis of the mRNA level of brain caspase-3 (**a**) and Bcl-2 (**b**). Data are presented as the means ± SD. ⁎, †, and ‡ denote significant change at *p* ≤ 0.05 versus control, BPA, and IR groups, respectively

Apoptosis is regulated by the protein caspase. As shown in Fig. 6, the expression of caspase-3 was significantly increased, whereas the expression of the anti-apoptotic marker Bcl-2 was significantly decreased in the BPA and IR groups in comparison to the control group. Meanwhile, the caspase-3 expression was downregulated and that of the Bcl-2 was upregulated upon NK supplementation.

**Fig. 7:**
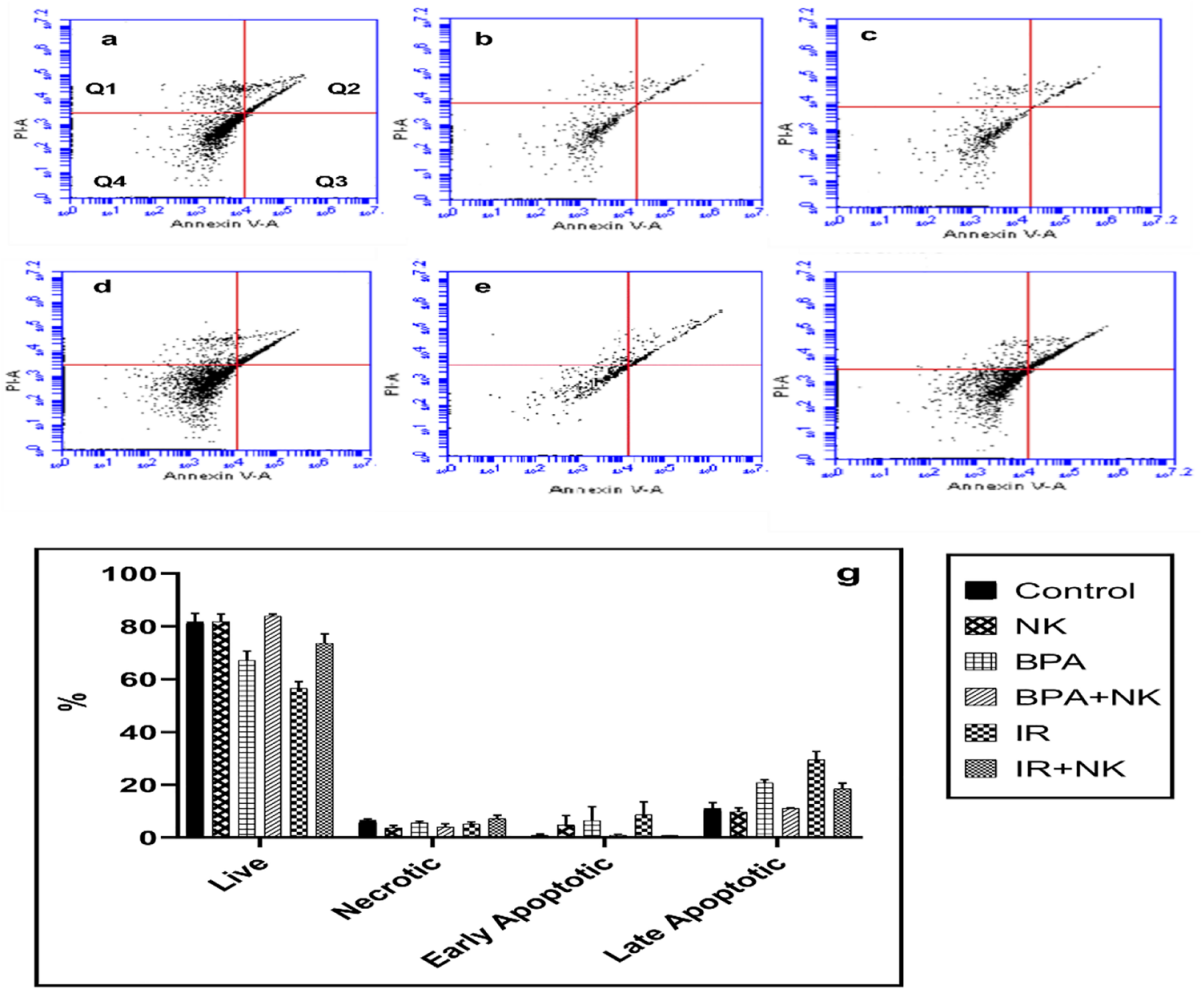
Dot plot chart of the percentages of live (Q4), necrotic (Q1), late apoptotic (Q2), and early apoptotic (Q3) cells representative of one sample, **a** control group, **b** NK group, **c** BPA group, **d** BPA + NK group, **e** IR group, and **f** I R + NK group. **g** Mean values of three samples of the percentages of live, early apoptotic, late apoptotic, and necrotic cells after injection of BPA or exposure to γ-radiation, and treatment with NK. FL1-H: a detector for fluorescence height for annexin V; FL2-H: a detector for fluorescence height for PI. Q1 represents necrosis, Q2 represents late apoptosis, Q3 represents early apoptosis, and Q4 represents live cells

Supporting these results, the results obtained from flow cytometry indicated that rats injected with BPA or exposed to γ-radiation showed a significant decrease in the live cells population accompanied by a significant increase in the percentages of early and late apoptotic and necrotic cells as compared with the control group. Conversely, treatment with NK increased the percentage of the live cells and decreased the percentages of late apoptotic cells as shown in Fig. 7.

### Histopathological studies

#### Liver tissue

**Fig. 8:**
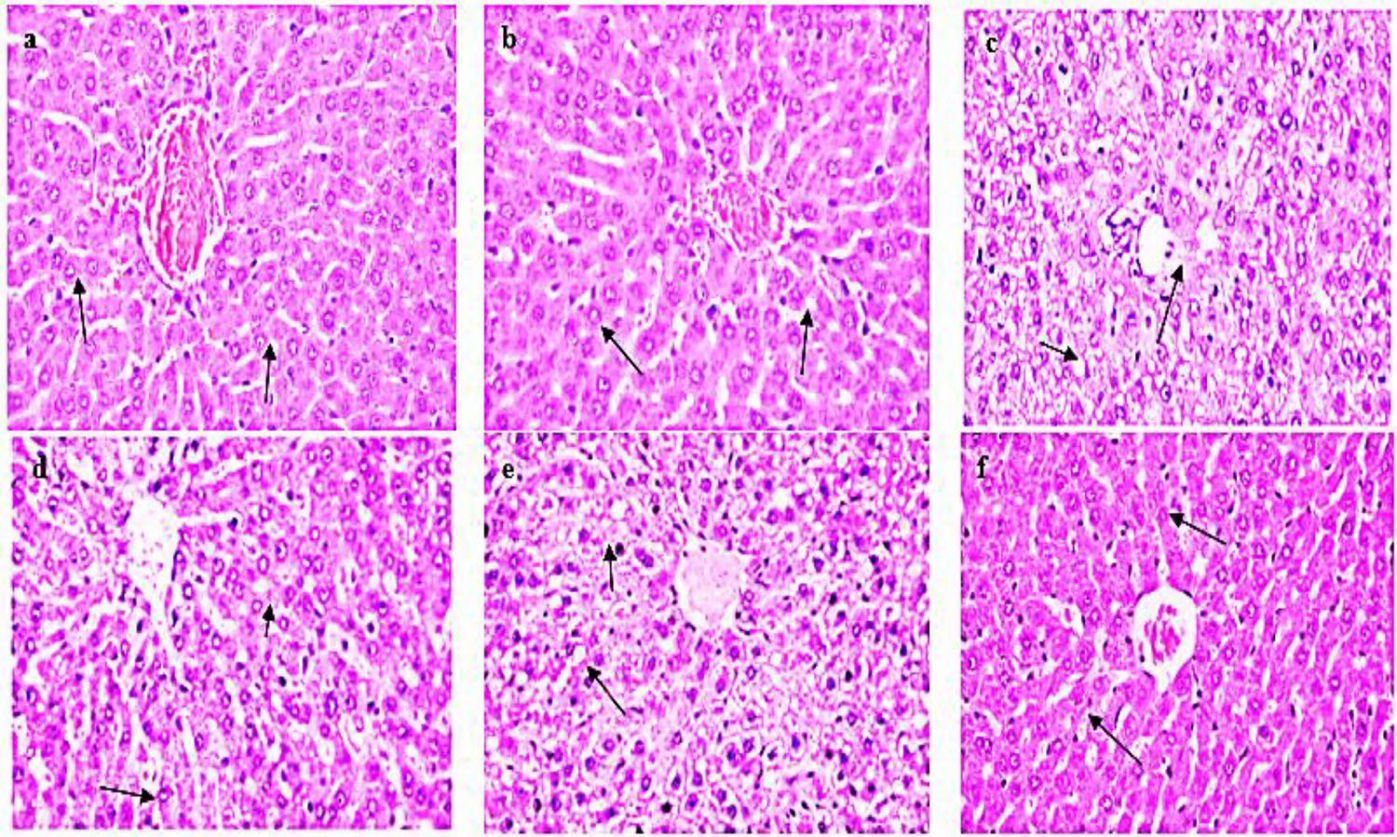
Photomicrograph of hepatic tissue section showing a normal histological structure of hepatic lobules in control group (**a**), strands of polygonal cells with prominent round nuclei, and eosinophilic cytoplasm (arrow) in NK group (**b**). BPA group (**c**): apoptosis of hepatocytes and intracellular fat droplets. BPA + NK group (**d**): ballooning degeneration of hepatocytes and narrowing of sinusoids with hyperplasia of Kupffer cells (arrow). IR group (**e**): few number hepatocytes with pyknotic nuclei and intracellular fat droplets. IR + NK group (**f**): mild swelling of hepatocytes and narrowing of hepatic sinusoids (arrow) (H&E × 200)

The liver tissue section of control and NK rats showed normal histological architecture of hepatocytes with a prominent central hepatic vein. Sinusoids are lined by a discontinuous layer of fenestrated endothelial cells with a fine arrangement of Kupffer cells (grade 0) (Fig. 8a, b).

The liver section of rats intoxicated with BPA showed severe effects ranging from ballooning degeneration with intracellular fat droplets of hepatocytes, especially in the centrilobular zone, coagulative necrosis, disorganization of hepatic cords, narrowing of hepatic sinusoids, and hyperplasia of Kupffer cells were also noticed (grade 4) (Fig. 8c). However, upon treatment of BPA with NK, the liver section showed ballooning degeneration of hepatocytes, narrowing of sinusoids with hyperplasia of Kupffer cells, large numbers of binucleated cells in the centrilobular zone in addition to mild to moderate swelling and narrowing of hepatic sinusoids (grade 2) (Fig. 8d). The hepatic tissue section of rats exposed to IR showed severe effects represented by disorganization of hepatic cords and necrobiotic changes of hepatocytes characterized by focal necrotic foci and hydropic degeneration of hepatocytes, some micro-vesicular steatosis and apoptotic bodies, nuclear pyknosis and granular cytoplasm of hepatocytes and narrowing of hepatic sinusoids and hyperplasia of Kupffer cells (grade 4) (Fig. 8e). Conversely, the liver tissue section of IR and NK rats showed mild swelling of hepatocytes and narrowing of hepatic sinusoids. Regenerated hepatic cells appeared deeply basophilic with marked hyperplasia of Kupffer cells were seen as grade 1 (Fig. 8f).

#### Brain tissue

**Fig. 9:**
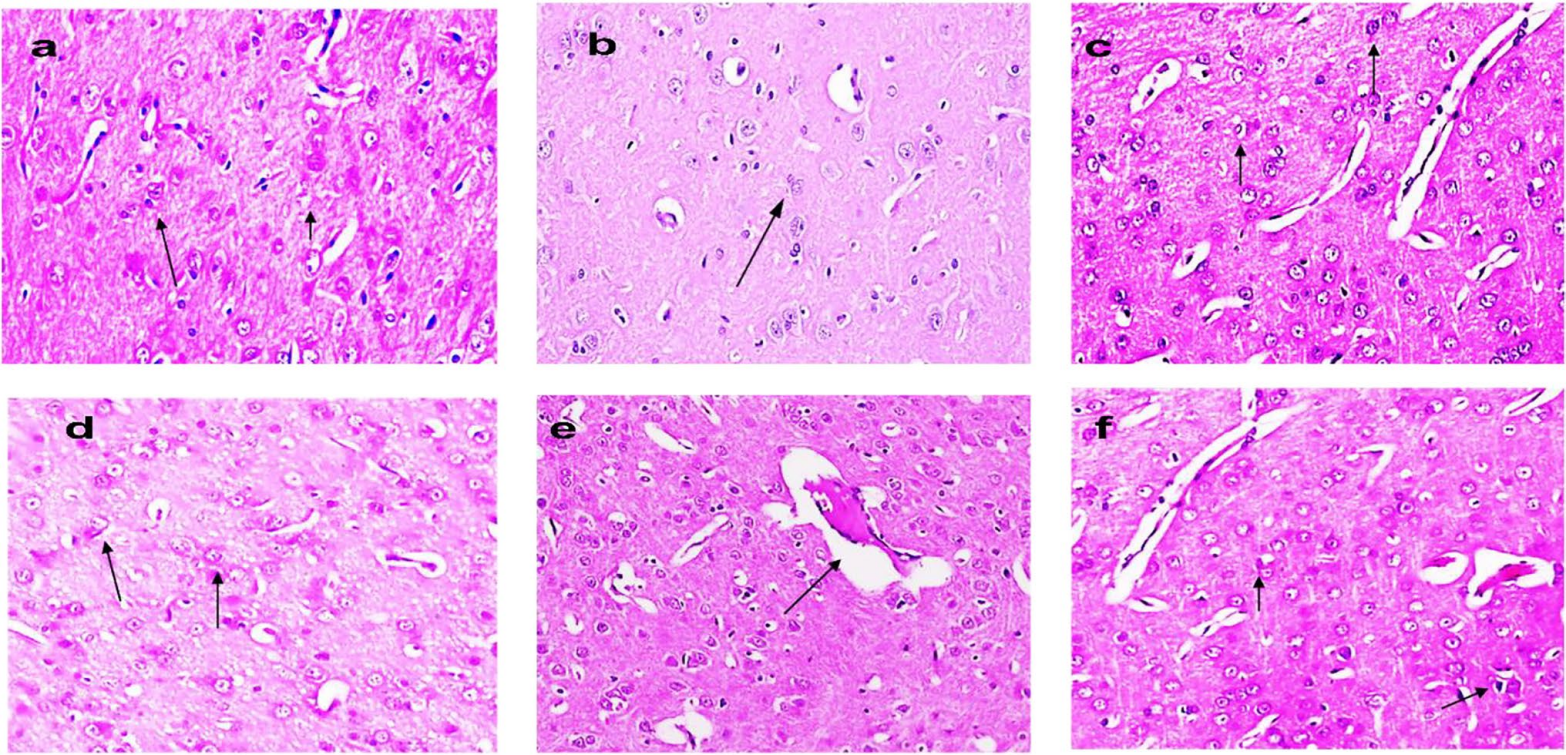
Photomicrograph of the cerebral cortex of control and NK groups (**a**, **b**) showing a normal arrangement of neuronal cells in association with small blood vessels in between (arrow) (H&E × 200). BPA: Photomicrograph of the cerebral cortex (**c**) showing eosinophilic apoptotic bodies and perivascular edema (arrow) (H&E × 200). BPA + NK: Photomicrograph of the cerebral cortex (**d**) showing few numbers of degenerated neuronal cells with pyknotic nuclei (arrow) (H&E × 200). IR: Photomicrograph of the cerebral cortex (**e**) showing eosinophilic apoptotic bodies and perivascular edema with congestion (arrow) (H&E × 200). IR + NK: Photomicrograph of the cerebral cortex (**f**) showing few numbers of apoptotic neuronal cells with focal gliosis (arrow) (H&E × 200)

**Fig. 10:**
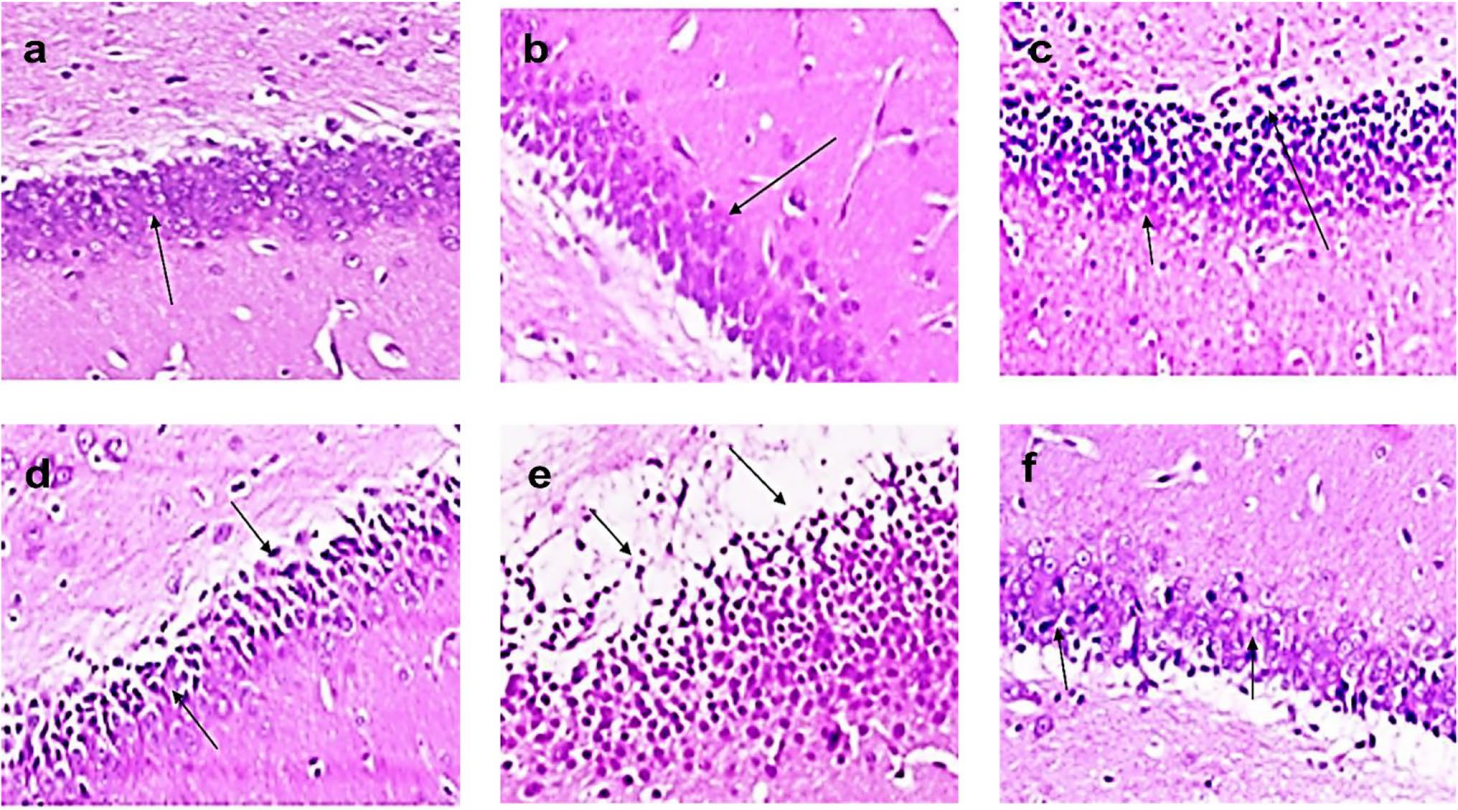
The photomicrograph of the hippocampus of the control and NK groups (**a**, **b**) showed the normal histological structure of compact granular cells with dark nuclei (arrow) (H&E × 200). BPA: Photomicrograph of the hippocampus (**c**) showing marked vacuolations of granular cell layers (arrow) (H&E × 200). BPA + NK: Photomicrograph (**d**) showing cellular organization and shrinkage in the size of pyramidal cells, with darkened nuclei in the hippocampus (arrow) (H&E × 200). IR: Photomicrograph (**e**) showing hippocampus cellular disorganization and shrinkage in the size of large pyramidal cells (arrow) (H&E × 200). IR + NK: Photomicrograph (**f**) showing small size of hippocampus large pyramidal cells, with darkened nuclei (arrow) (H&E × 200)

The brain sections of the control and NK groups display the normal architecture of the brain consisting of the cerebral cortex, cerebral (neuronal cells with oval or rounded nuclei arranged with no sharp boundaries in association with small blood vessels in between) (Fig. 9a and b), and hippocampus which appeared as layers of compact granular cells with dark nuclei (Fig. 10a and b). However, the injection of BPA resulted in moderate neuronal degeneration and apoptosis of some neuronal cells which is associated with focal gliosis and satellitosis, neuronophagia, and perivascular edema in the cerebral cortex, score (+ +) (Fig. 9c). Additionally, cellular disorganization, shrinkage in the size of large pyramidal cells, with darkened nuclei, and marked vacuolation in the granular cell layers were observed in the hippocampus (Fig. 10c). In contrast, the co-administration of NK decreased the numbers of degenerated neuronal cells, ameliorated gliosis, satellitosis, and neuronophagia in the cerebral cortex (mild effect), score ( +) (Fig. 9d) along with improving the hippocampal cellular organization (Fig. 10d). Similarly, the exposure of rats to γ-radiation resulted in severe cerebral cortex neuronal degeneration (darkly stained surrounded by per-cellular haloes spaces), apoptosis of neuronal cells (eosinophilic bodies associated with focal gliosis and satellitosis), and marked perivascular edema and congestion in the cerebral blood vessels, score (+ + +) (Fig. 9e). Additionally, cellular disorganization, shrinkage in the size of large pyramidal cells, with darkened nuclei, and marked vacuolation in the granular cell layers were observed in the hippocampus (Fig. 10e). However, NK supplementation decreased the apoptotic neuronal cells, gliosis, satellitosis, and the perivascular edema, score (+ +) (Fig. 9f) with a slight improvement in the hippocampal cellular organization, pyramidal cells, and the granular cell layers (Fig. 10f).

## Discussion

The current study showed that exposure to the environmental toxicants BPA and γ-radiation causes multi-organ toxicity through several mechanisms such as impairment of oxidative status, signaling pathways, and hepatic and neuronal functions as well as disruption of the inflammatory responses (Ma et al. 2019; Bolbol et al. 2021).

The liver is one of the most radiosensitive organs (Kim et al. 2017) where the BPA is metabolized to form BPA-glucuronide which is accumulated in the liver leading to disturbing liver integrity. Herein, the intoxication with BPA resulted in hepatic damage evidenced by the impaired activity of liver enzymes along with the reduced levels of serum total protein and confirmed with the histopathological examinations. Our results are parallel to that of Kamel et al. (2018) and Lebda et al. (2020), who ensured the toxic effects of BPA on the liver via disrupting the liver tissue architecture and increasing the permeability of the hepatocellular plasma membrane leading to the leakage of the liver enzymes to the blood and elevated activities of ALT, AST, and levels of ALP. Additionally, our results are in accordance with that of El-Shahat et al. (2021) who reported that the rise in the serum transaminases activities and alkaline phosphatase after γ-irradiation may be due either to the release of enzymes from radiosensitive tissues or to changes in its synthesis and may be related to the extensive breakdown of liver parenchyma. However, co-administration of nattokinase significantly ameliorates the liver function by reducing the activities of ALT and AST as well as the levels of ALP (Hideaki Suzuki et al. 2017).

Furthermore, impaired hepatic function and loss of liver ability to detoxify toxins are associated with lower levels of total protein and higher levels of ammonia. This agrees with Abdelgawad et al. (2019) and El-Baz et al. (2021) who indicated that impaired liver function decreases the total protein and elevates the levels of ammonia in the blood that crosses the blood-brain barrier and is metabolized in the brain astrocytes to glutamine. Indeed, ammonia interacts with other factors to disturb brain function (Butterworth 2019).

Both gamma irradiation and BPA-mediated neurotoxicity via several mechanisms, including production of reactive oxygen species (ROS), mitochondrial degeneration, neuroinflammation, and protein degradation, lead to apoptotic cell death (Wang et al. 2017; Sharma et al. 2019). The brain is very sensitive to oxidative stress and ROS due to the extensive consumption of oxygen and high content of polyunsaturated fatty acids along with low antioxidant levels (Li et al. 2013; Cheignon et al. 2018).

In the present study, consistent with the proposed mode of action, the exposure to BPA or γ-irradiation was accompanied by significant deleterious changes in antioxidant status, as a response to the induced oxidative stress and production of ROS. In the present study, an increase in the levels of lipid peroxidation and a decline in the levels of GSH were noticed in the brain tissues. These results are consistent with El Morsy and Ahmed (2020) who revealed that BPA intoxication disturbed the brain’s redox state which can easily penetrate the blood–brain barrier and induce its toxicity through oxidative stress. Furthermore, Kale et al. (2019) reported that radiation increased lipid peroxidation in the brain especially neuronal cells via ROS production, thus impairing both the structure and function of neurons.

Previous studies had shown that oxidative stress promotes Aβ aggregation and tau hyperphosphorylation leading to neuronal toxicity and injury as well as impaired cognitive function (Khandelwal et al. 2020). The accumulation of both Aβ and phosphorylated tau is one of the main pathological hallmarks of Alzheimer’s disease. Moreover, the disruption of the cholinergic system is accompanied by cognitive impairments in neurodegenerative diseases (Bensalem et al. 2016). Herein, both BPA and IR resulted in brain toxicity and impaired cognitive functions manifested through the higher levels of the misfolded proteins Aβ and p-tau besides the decline in the acetylcholine levels (ACh). Our results are in harmony with that of Hawas et al. (2020) who indicated that γ-radiation reduces ACh and increases Aβ in the brain. Li et al. (2014) demonstrated that oxidative stress induced by ionizing radiation mediates the increased tau phosphorylation. Moreover, Sharma et al. (2018) showed that high-dose radiation promotes misfolding and aggregation of proteins involved in the progression of the neurodegenerative disease. This may be due to neural loss and demyelination, vascular abnormalities and changing the brain microenvironment consequently, cognitive defects and impaired neurogenesis. Additionally, our results are consistent with that of Wang et al. (2017) who showed that BPA enhanced the level of Aβ and the expression of phosphorylated tau and Sukjamnong et al. (2020) who reported that maternal exposure to BPA disrupts brain functions and induces cognitive deficits and learning-memory impairment in offspring mice. Administration of BPA leads to the death of cerebellar granule cells, olfactory bulb neurons, and basal forebrain cholinergic neurons. Furthermore, BPA causes choline toxicity, resulting in decreased acetylcholine transferase activity. This demonstrates that administration of BPA induced the death of forebrain cholinergic neurons leading to a reduction of the acetylcholine and cognitive dysfunction (Mahdavinia et al. 2019).

Conversely, supplementation with nattokinase markedly declined the levels of Aβ and p-tau and alleviated the levels of acetylcholine. These results are parallel to that of Bhatt et al. (2018) and Chen et al. (2018) who reported that treating rats suffering from Alzheimer’s disease (AD) with nano-nutraceuticals containing NK ameliorated the impaired cognitive functions and enhanced the clearance of Aβ along with the inhibition of the BACE-1 activity, thus suggesting a neuroprotective efficacy of NK. Moreover, Fadl et al (2013) showed that NK supplementation treats AD via modulating brain acetylcholinesterase activity and IL-6 levels. Additionally, Wang et al. (2012) reported that the neuroprotective effects of NK were through its ability to scavenge the free radicals.

Interestingly, the accumulation of Aβ aggregates in the brain tissues activates non-neuronal cells, especially microglia cells and astrocytes that mediate neuroinflammation. These activated cells release inflammatory cytokines causing impaired neurogenesis and increased susceptibility to neurodegenerative disorders such as AD (Heneka et al. 2015; Kinney et al. 2018). Moreover, the astrocytes (the major glial cells) were reactivated through the overexpression of an intermediate filament protein glial fibrillary acidic protein (GFAP) (Schiffer et al. 1986; Nahirnyj et al. 2013). Consequently, the upregulated expression of the GFAP during neuroinflammation may be a reliable marker of brain injury.

The overproduction of oxidative stress activates the nuclear factor kappa B (NF-ĸB) a transcription factor involved in various processes such as apoptosis and inflammation, therefore, the release of various pro-inflammatory cytokines (Kaulmann and Bohn 2014; Ahmed et al. 2017). Accordingly, in response to the intoxication with the BPA or the exposure to γ-radiation, the present study showed marked upregulated levels of the neuroinflammatory mediators IL-6 and NF-ĸB and GFAP along with the abolished levels of the anti-inflammatory marker IL-10. Our data are in line with that of Acaroz et al. (2019) who reported that BPA induced neuroinflammation via the overexpression of the brain pro-inflammatory cytokines (IL-1β and IL-6) and decreased anti-inflammatory IL-10. Additionally, Zhu et al. (2015) showed that BPA significantly upregulated the phosphorylated NF-κB p65 levels as well as the GFAP levels confirming the involvement of the microglial cells and NF-κB in the inflammatory response and impaired neurogenesis after BPA intoxication (Tiwari et al. 2015). Moreover, radiation triggers neuroinflammation and significantly increased the brain levels of the pro-inflammatory mediators IL-1β, IL-6, and NF-κB (Lee et al. 2010; Yang et al. 2017). Besides, the astrocytes proliferate and exhibit hypertrophic nuclei/cell bodies with an apparent elevation in the GFAP expression upon radiation injury (Greene-Schloesser et al. 2012; Gao et al. 2021).

Nrf2/HO-1 signaling pathway is an important endogenous protective system for multi-organ against different stimuli and environmental stressors. It is involved in the anti-inflammation, anti-oxidative, and antiapoptotic response and thus can be targeted for the treatment of the diseases linked to oxidative stress and inflammation (Li et al. 2020). Nrf2 and NF-ĸB signaling pathways interact to regulate the function of downstream target proteins. Previous studies reported that the depletion of Nrf2 promotes the NF-κB–mediated proinflammatory reactions (Ahmed et al. 2017). Based on the aforementioned data, our results revealed that BPA or γ-irradiation concomitant with oxidative stress and neuroinflammation, there is a remarkable suppression in the gene expression of both Nrf2 and HO-1. These results work in with that of Chiang et al (2022) who observed downregulation of the Nrf2 and HO-1 gene transcription upon intoxication with BPA. Moreover, Zhang et al. (2021) demonstrated that irradiation impedes the protein and gene expression of the Nrf2 and HO-1 via ROS the main mediators of radiation-induced damage, therefore, inducing cell apoptosis and stimulating numerous inflammatory factors.

According to the previous studies, the impaired oxidative status and overproduction of ROS by both BPA and IR trigger mitochondrial dysfunction and promote the mitochondrial apoptotic signaling pathway. In addition to the results from flow cytometry, this was evidenced through the upregulation of caspase-3 expression accompanied by the downregulation of the antiapoptotic Bcl-2 gene expression. Accordingly, BPA and IR promote neurotoxicity, neuronal degeneration, and apoptosis (Huang et al. 2018; El Morsy and Ahmed 2020).

Contrarily, the co-treatment with NK significantly increased the anti-inflammatory IL-10 levels, upregulated the expression of Nrf2 and its downstream target HO-1 as well as the antiapoptotic Bcl-2 expression with concomitant suppression of the proinflammatory mediators IL-6 levels, NF-ĸB, GFAP expression, and the apoptotic caspase-3 activity. The anti-inflammatory and anti-apoptotic effects of NK coincided with the results of Bhatt et al. (2018) and Gallelli et al. (2021) who confirmed that NK inhibits the pro-inflammatory IL-6 and elevates the anti-apoptotic Bcl-2 activity, therefore, protecting neurons from apoptosis and ameliorates the neurological diseases. Moreover, NK notably declined the GFAP expression consequently, alleviating the neuroinflammation by switching the proinflammatory microglia into an anti-inflammatory (Huang et al. 2021). Additionally, NK attenuated ischemia–reperfusion injury and improved neurological function via the activation of the Nrf2/HO-1 axis (Loboda et al. 2016). Furthermore, Li et al. (2020) confirmed that modulation of the Nrf2/HO-1 signaling pathway ameliorates neuronal damage and inhibits the oxidation tension contributing to inflammation and apoptosis.

## Conclusion

In conclusion, our results showed that NK extraordinarily enhanced the impaired oxidative status along with the clearance of misfolded proteins. Furthermore, NK alleviated the neuroinflammation via suppressing the NF-κB, modulating Nrf2/HO-1 pathway and astrocyte/glial cell activation. Additionally, NK ameliorated both neuropathology and neurological function by improving the cholinergic deficits. Collectively, NK may be used to protect the body not only from hepatic damage but also from neurotoxicity and neurodegenerative diseases derived from environmental toxicants. However, further studies are required to determine the exact underlying mechanisms.

## Data Availability

All data obtained from this study are included in the current manuscript.
