# Correction to: Nattokinase attenuates bisphenol A or gamma irradiation-mediated hepatic and neural toxicity by activation of Nrf2 and suppression of inflammatory mediators in rats

**DOI:** 10.1007/s11356-026-37513-5

**Published:** 2026-02-18

**Authors:** Mustafa M. M. Elbakry, Somaya Z. Mansour, Hamed Helal, Esraa S. A. Ahmed

**Affiliations:** 1 Biochemistry Department, Faculty of Science, Ain Shams University, Cairo, Egypt; 2 Radiation Biology Research, National Center for Radiation Research and Technology, Egyptian Atomic Energy Authority, Nasr City, Cairo, 11787 Egypt; 3 Zoology Department, Faculty of Science, Al-Azhar University, Cairo, Egypt

**Correction to: Environmental Science and Pollution Research**

10.1007/s11356-022-21126-9

The authors sincerely apologize for the unintended errors which occurred while uploading the figures (***Figure 7 and Figure 9)*** to the journal platform during the article publication.

- ***In Figure 7, the incorrect image was uploaded in error for Figure 7c***
- ***In Figure 9, the incorrect image was uploaded in error for Figure 9f***

As a corrective action to solve such concern, we attached the correct figures (***Figure 7 and Figure 9***) that can be uploaded instead of the previously uploaded figure.

We appreciate your kindness and please accept the authors' apology for that accidental mistake.

**Figure:**
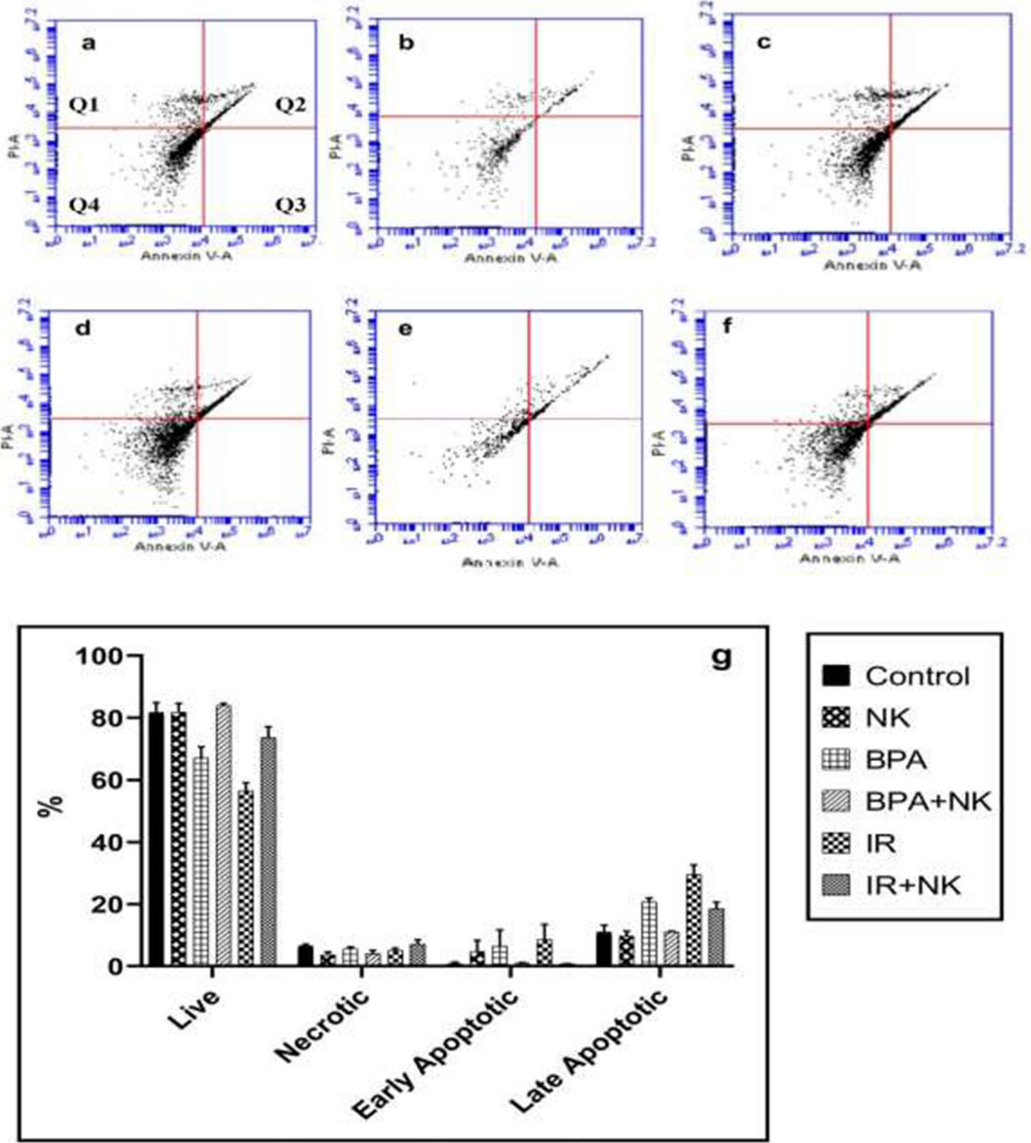
**Fig. 7** Dot plot chart of the percentages of live (Q4), necrotic (Q1), late apoptotic (Q2), and early apoptotic (Q3) cells representative of one sample, a control group, b NK group, c BPA group, d BPA+ NK group, e IR group, and f I R+ NK group. g Mean values of three samples of the percentages of live, early apoptotic, late apoptotic, and necrotic cells after injection of BPA or exposure to γ-radiation, and treatment with NK. FL1-H: a detector for fluorescence height for annexin V; FL2-H: a detector for fluorescence height for PI. Q1 represents necrosis, Q2 represents late apoptosis, Q3 represents early apoptosis, and Q4 represents live cells

**Figure:**
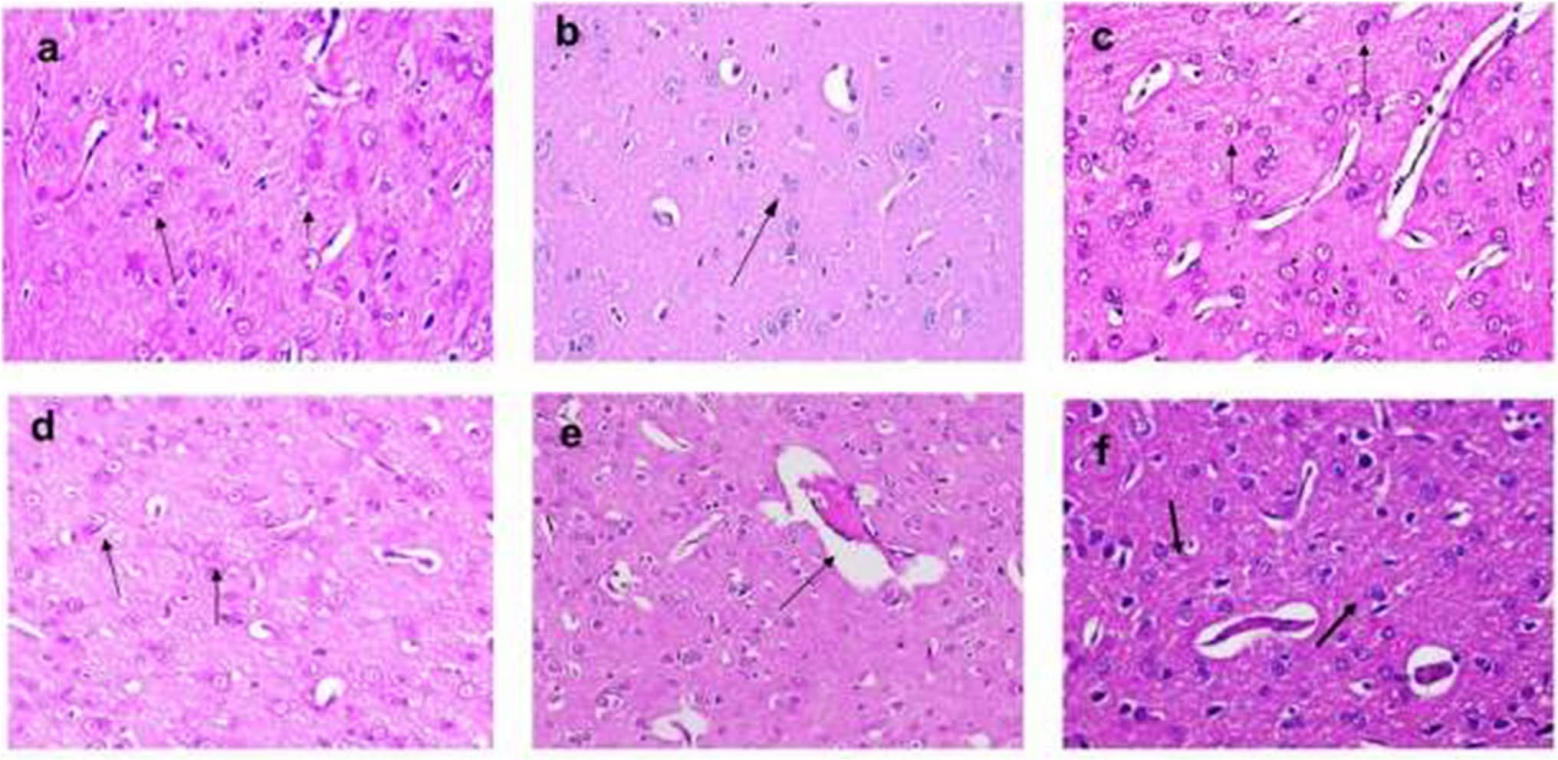
**Fig. 9** Photomicrograph of the cerebral cortex of control and NK groups (**a**, **b**) showing a normal arrangement of neuronal cells in association with small blood vessels in between (arrow) (H&E × 200). BPA: Photomicrograph of the cerebral cortex (**c**) showing eosinophilic apoptotic bodies and perivascular edema (arrow) (H&E ×200). BPA+ NK: Photomicrograph of the cerebral cortex (**d**) showing few numbers of degenerated neuronal cells with pyknotic nuclei (arrow) (H&E ×200). IR: Photomicrograph of the cerebral cortex (**e**) showing eosinophilic apoptotic bodies and perivascular edema with congestion (arrow) (H&E× 200). IR+ NK: Photomicrograph of the cerebral cortex (**f**) showing few numbers of apoptotic neuronal cells with focal gliosis (arrow) (H&E×200)

